# 

**Published:** 1980

**Authors:** 


### BRISTOL MEDICO-CHIRURGICAL JOURNAL

JULY / OCTOBER 1980
VOLUME 95 (iii iv)
NUMBER 355 356

NATIONAL

LIBRARY

S 13 APR 1982 Z

OF

MEDICINE

